# Further resolution of the house mouse (*Mus musculus)* phylogeny by integration over isolation-with-migration histories

**DOI:** 10.1186/s12862-020-01666-9

**Published:** 2020-09-15

**Authors:** Megan Phifer-Rixey, Bettina Harr, Jody Hey

**Affiliations:** 1 Department of Biology, Monmouth University, West Long Branch, NJ USA; 2 Department of Evolutionary Genetics, Max-Planck-Institute for Evolutionary Biology, Plön, Germany; 3 Department of Biology, Center for Computational Genetics and Genomics, Temple University, Philadelphia, PA USA

**Keywords:** Speciation, Divergence, Population size, IMa3

## Abstract

**Background:**

The three main subspecies of house mice, *Mus musculus castaneus, Mus musculus domesticus*, and *Mus musculus musculus,* are estimated to have diverged ~ 350-500KYA. Resolution of the details of their evolutionary history is complicated by their relatively recent divergence, ongoing gene flow among the subspecies, and complex demographic histories. Previous studies have been limited to some extent by the number of loci surveyed and/or by the scope of the method used. Here, we apply a method (IMa3) that provides an estimate of a population phylogeny while allowing for complex histories of gene exchange.

**Results:**

Results strongly support a topology with *M. m. domesticus* as sister to *M. m. castaneus* and *M. m. musculus*. In addition, we find evidence of gene flow between all pairs of subspecies, but that gene flow is most restricted from *M. m. musculus* into *M. m. domesticus*. Estimates of other key parameters are dependent on assumptions regarding generation time and mutation rate in house mice. Nevertheless, our results support previous findings that the effective population size, *N*_*e,*_ of *M. m. castaneus* is larger than that of the other two subspecies, that the three subspecies began diverging ~ 130 - 420KYA, and that the time between divergence events was short.

**Conclusions:**

Joint demographic and phylogenetic analyses of genomic data provide a clearer picture of the history of divergence in house mice.

## Background

The house mouse (*Mus musculus*) has long been a genetic model for human biology and disease (reviewed in [1–3]). House mice have also grown into a model system for evolutionary genetics, fueling investigations of topics ranging from meiotic drive to adaptive introgression (e.g. [4, 5]). In particular, studies in house mice have shed light on the process of speciation and the genetic basis of reproductive isolation (e.g. [6–14]). More recently, studies have leveraged the increasing geographical distribution of house mice to investigate the genetics of phenotypic change and adaptation accompanying range expansion (e.g. [15, 16]). However, much of this work relies on an understanding of the evolutionary history of house mice that continues to be refined.

The most numerous subspecies of house mice, *Mus musculus domesticus*, *Mus musculus musculus*, and *Mus musculus castaneus*, are found over different, but overlapping, geographical ranges (reviewed in [17]). While the subspecies can be crossed in the lab, in some cases, hybrid males are sterile or have reduced fertility (e.g., [7, 18, 19]). There is extensive evidence of hybridization between the subspecies in the wild. The hybrid zone between *M. m. domesticus* and *M. m. musculus* has been particularly well-studied (for review, see [20]), but *M. m. domesticus* in the United States harbor introgression from *M. m. castaneus* [21] and there is evidence of a hybrid zone in China between *M. m. castaneus* and *M. m. musculus* [22]. In fact, evidence suggests that another subspecies in the group, *Mus musculus molossinus*, found in Japan, was formed by hybridization between *M. m. castaneus* and *M. m. musculus* [23].

The group is believed to have originated in Southwestern Asia [24, 25] and analyses support a near simultaneous divergence between the three subspecies within the last ~ 350,000–500,000 years (e.g., [25–28]). The demographic histories of the subspecies differ markedly. The center of diversity for *M. m. castaneus* is also in Southwestern Asia and estimates of effective population size (*N*_*e*_) of *M. m. castaneus* are large (~ 200,000-700,000 [27–29]). On the other hand, estimates of *N*_*e*_ for *M. m. domesticus* (~ 58,000-200,000) and *M. m. musculus* (25,000-120,000 [27, 28, 30]) are much smaller. Both are believed to have undergone bottlenecks as they shifted their ranges—*M. m. domesticus* through the Middle East and North Africa into Western Europe and *M. m. musculus* into North Asia and Eastern Europe [25, 26].

The combination of a relatively recent divergence, ongoing gene flow, and complex demography complicates the resolution of the phylogenetic relationships between the subspecies. Nevertheless, previous studies have provided fundamental insights into the history of their divergence. Geraldes et al. [27] and Geraldes et al. [28] sampled many individuals of all three subspecies (*n* = 26–60) across a modest number of loci (8 and 27 respectively) and used multiple two-population isolation-with-migration (IM) models [31–33] to estimate key parameters, e.g., *N*_*e*_, migration rates, and divergence times. However, resolution of the topology of the subspecies group using a pairwise approach was not possible and resolution of divergence times was limited. In some instances, it was not possible to obtain either a reliable estimate of divergence time or confidence intervals for estimates of divergence time. In other analyses, confidence intervals were large [27, 28]. Suzuki et al. [25] found more topological resolution by applying phylogenetic methods to mtDNA from a sample of mice spanning Europe, Asia, Africa, and Australia. Their results support *M. m. domesticus* as sister to *M. m. castaneus* and *M. m. musculus*, and provide evidence for a split between *M. m. domesticus* and (*M. m. castaneus*, *M. m. musculus*) ~459KYA (CI:~ 325 - 481KYA). However, reliance on mtDNA alone can be problematic when estimating phylogenies (e.g., [34]). A different approach, Bayesian concordance analysis, uses genome wide data from single individuals of each subspecies to estimate gene trees. Two such studies have considered the topology of the house mouse subspecies [35, 36]. Both find support for *M. m. castaneus* and *M. m. musculus* as sister to *M. m. domesticus,* but the alternate topologies have moderate support, highlighting extensive phylogenetic discordance in the group. For example, in White et al. [36] only 39% of gene trees supported the primary topology compared to the 33% expected under a simultaneous divergence. In addition, this approach is not useful for estimating other parameters of interest.

Here, we revisit the history of divergence among the subspecies by taking advantage of a new method that allows for phylogeny estimation with multiple species or populations that is especially useful in this potential trichotomy (IMa3 [37];). IMa3 is a genealogy sampling program [38] that implements a multi-population IM model with a novel “hidden genealogy” Markov-chain Monte Carlo (MCMC) update that permits the sampling of population phylogenies [37]. Once an estimate of the posterior probability distribution of population phylogenies has been obtained, the program can be run a second time, while fixing the phylogeny on the estimated value, in order to obtain estimates for *N*_*e*_ values, migration rates and branch lengths (splitting times). With these two successive runs, the method provides for a joint estimate of the rooted population phylogeny and the complex demographic history within that phylogeny [37]. We applied this approach to publicly available genomic data, incorporating 200 randomly selected autosomal loci for multiple individuals of each subspecies ([39]; Table S1). Our results strengthen and refine our understanding of divergence among house mouse subspecies. Most notably, we found strong support for a sister relationship between *M. m. musculus* and M. m. *castaneus* and that divergence among the three species likely began no more than ~500KYA and possibly as recently as ~130KYA.

## Results and discussion

**Fig. 1:**
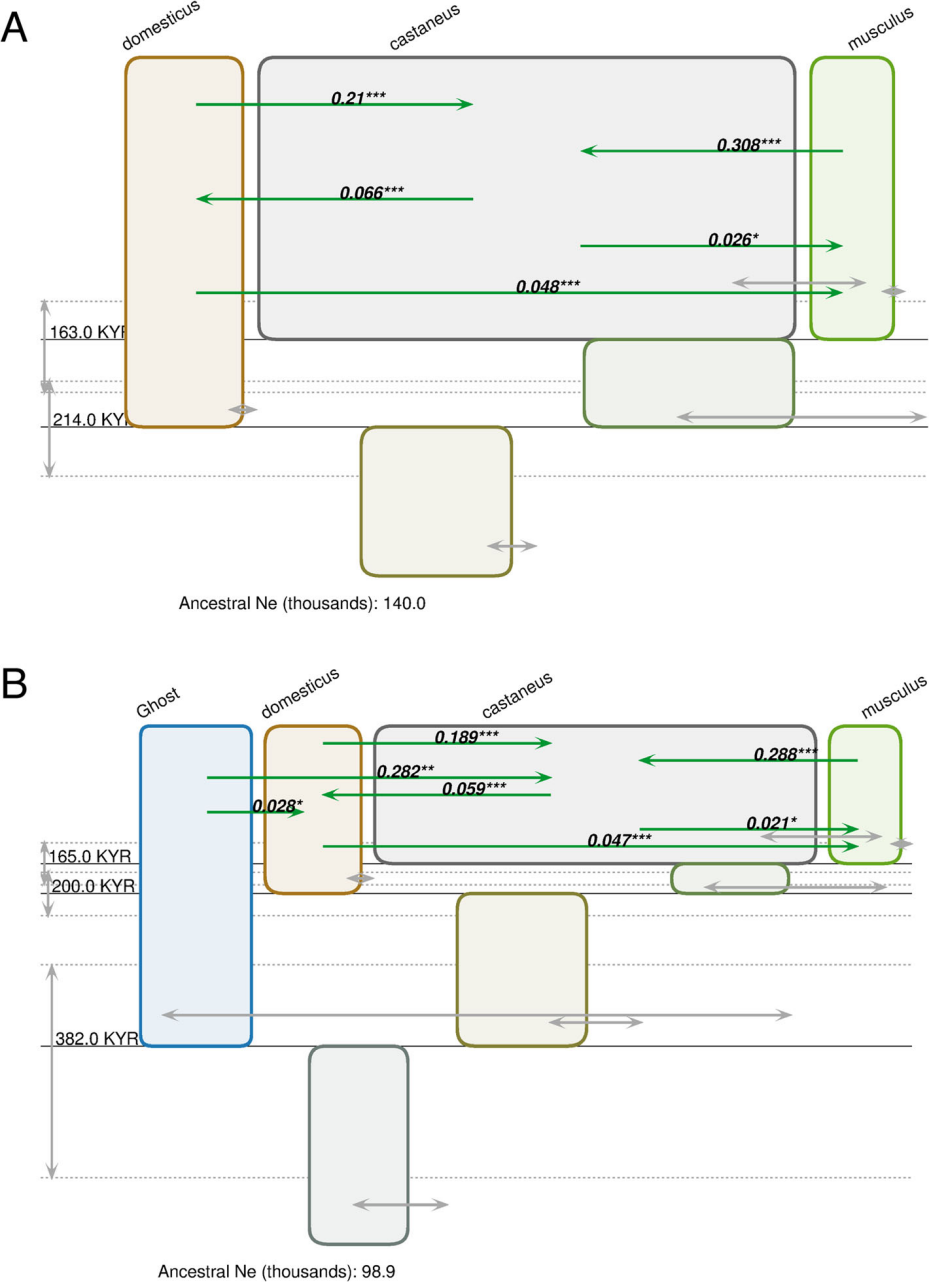
Representations of estimated IM models generated by IMa3 and the IMfig program [37] for the three subspecies of house mouse. The phylogeny is depicted as a series of boxes organized hierarchically, with ancestor boxes positioned in between the corresponding descendants, and the width of boxes proportional to estimated *N*_*e*_. Gray arrows extending to the left and right of the right boundary of each population box depict 95% confidence intervals for *N*_*e*_ values. Splitting times are depicted as solid horizontal lines, with text values on the left. Confidence intervals for splitting times are shown as vertical gray arrows on the left, and parallel dashed lines. Migration arrows (if shown) indicate estimated 2 *Nm* values from one population to another over the time interval when both populations exist. Arrows are shown only for estimated migration rates that are statistically significant at or above the 0.05 level (^*^p < 0.05, ^**^p < 0.01, ^***^p < 0.001 [33]). (**a**) without a ghost population (**b**) with a ghost population included. Estimates assume 1.5 generations/year and 6 × 10^−9^ mutations per base pair per generation

IMa3 analysis of 200 randomly selected autosomal loci supported a phylogeny with *M. m. musculus* and *M. m. castaneus* as sister to *M. m. domesticus,* with or without the inclusion of an unsampled ‘ghost’ population (Fig. 1, Table 1). Without a ghost population, the posterior probability for that topology was 0.759 compared to 0.146 for a sister relationship between *M. m. castaneus* and *M. m. domesticus*, and 0.096 for a sister relationship between *M. m. musculus* and *M. m. domesticus.* With a ghost population, the posterior probability for that topology was 0.921 compared to 0.050 for a sister relationship between *M. m. castaneus* and *M. m. domesticus*, and 0.029 for a sister relationship between *M. m. domesticus and M. m. musculus.* Overall, there is little evidence that an unsampled population has shaped the demographic history of these three subspecies, given that the estimates of the posterior distribution of phylogenies, as well as parameter estimates (Table 1; Tables S2, S3, S4, S5, S6 and S7), are similar in runs that did and did not include a ghost population.

**Table 1:** The topology that places *M. m. castaneus* sister to *M. m. musculus* was sampled most frequently, whether a ghost population was included or not. The three subspecies are numbered from 0 to 2 (corresponding to *M. m. castaneus*, *M. m. domesticus*, and *M. m. musculus,* respectively). Ancestral populations are numbered beginning with 3 and are ordered numerically in time (i.e. 4 is the ancestor of all populations). When a ghost population is included, it is an outgroup to the sampled subspecies and has not been included in the species tree notation

Model	Species Tree	Count	Frequency
Without ghost	(1,(0,2)3)4	237,697	0.758636
	(2,(0,1)3)4	45,610	0.145753
	(0,(1,2)3)4	29,919	0.095610
With ghost	(1,(0,2)3)4	227,602	0.920739
	(2,(0,1)3)4	12,363	0.050013
	(0,(1,2)3)4	7230	0.029248

To convert parameter estimates, which are scaled by mutation rate [31], to useful demographic scales, a mutation rate and generation time are required. We assumed 1.5 generations per year, which is intermediate between reported frequencies of one and two generations per year [27, 40]. For mutation rate, we used a recent estimate based on trio sequencing of 6 × 10^−9^ mutations per base pair per generation [41]. This is similar to a previous phylogeny-based estimate of 4.2 × 10^−9^ [42] and to an autosomal estimate from [27] (4.1 × 10^−9^). For clarity, unless otherwise noted, we report scaled estimates based on these assumptions. However, for more direct comparison to previous studies, we also provide estimates scaled using 1 and 2 generations/year and 4.1 × 10^−9^ mutations per base pair per generation (Tables S2, S3, S4, S5 and S6).

Estimates of *N*_*e*_ for each of the subspecies were consistent with previous work (Tables S5, S6 [27–30]). Given our assumptions for mutation rate and generation time, *M. m. castaneus* had the largest estimated *N*_*e*_, with confidence intervals from both models (with and without a ghost population) ranging from ~ 419,000-569,000. *M. m. domesticus* was intermediate, with estimates ranging from ~ 91,000–121,000 and the smallest estimates of *N*_*e*_ were from *M. m. musculus*, ~ 67,000–86,000 (Table S5-S6). As with previous studies, estimates of ancestral *N*_*e*_ were of the same order of magnitude as estimates for the extant subspecies (without ghost: 139,951 CI: 118,420-163,709; with ghost: 105,347 CI: 41,320-147,783; Table S5, S6 [27, 28]).

For all migration rate parameters, in both directions between pairs of subspecies, the 95% confidence interval for estimates of migration did not include zero. In addition, estimates of migration rate were statistically significant, whether including a ghost population or not, with the exception of migration from *M. m. musculus* into *M. m. domesticus* (Fig. 1; Tables S2, S3, S7). In some cases, such as gene flow into *M. m. castaneus* from the other two subspecies, the estimated population migration rate (*2 Nm)* was quite high (e.g., 0.210 and 0.308 from *M. m. domesticus* and *M. m. musculus*, respectively, in the analysis without a ghost population, Table S7). While estimates of migration rates were generally reported as not statistically significant in previous studies, patterns of gene flow inferred here are broadly consistent with previous findings that reject a model of speciation with no gene flow and suggest more migration into *M. m. castaneus* than into either of the other two subspecies [27, 28]. A model of speciation with reciprocal migration, but that is more limited into *M. m. domesticus* and *M. m. musculus,* is also consistent with results from studies of contemporary hybrid zones, laboratory crosses, and genome-wide patterns of genetic variation. Hybridization occurs between each pair of subspecies in the wild (e.g., [22, 24, 26, 43]), but the degree of reproductive isolation observed in the lab differs. Crosses between *M. m. domesticus* and *M. m. musculus* can result in significant male sterility (e.g., [13, 44]), while impacts on male fertility are not observed until the F_2_ in crosses between *M. m. castaneus* and *M. m. domesticus*, [19] and have not been reported at all in crosses between *M. m. castaneus* and *M. m. musculus*. Levels of genetic differentiation between *M. m. musculus* and *M. m. domesticus* are also higher than between any other pair of subspecies (e.g., [11, 28]).

The estimated history given the dominant phylogeny and our assumptions regarding mutation rate and generation time suggests all three subspecies diverged within the last 250,000 years (Fig. 1; Tables S2, S3 and S4). Divergence between *M. m. domesticus* and (*M. m. castaneus*, *M. m. musculus*) was estimated at 214,158 KYA (CI: 188,767-243,559) and the subsequent divergence between *M. m. castaneus*, *M. m. musculus* was estimated at 163,375 KYA (CI: 142,661-194,781). Including a ghost population shifted these estimates slightly to 200,010 KYA (CI: 175,888-227,482) and 165,167 KYA (CI: 141,716-189,959), respectively. Our main analysis was limited to runs with a maximum of 200 intergenic loci because of time and MCMC mixing constraints. One benefit of increasing genomic and computational resources is that we can assess the effect of sampling on our results using a separate analysis with 200 different loci, sampled at random using the same protocol as the primary set. The phylogeny estimates and the IM model estimates with this second set of 200 randomly selected loci were very similar to the primary analysis (Table S8, S9, S10 and 11 and Figure S1).

While these estimates are more recent than reported in most previous studies, it is important to note that estimates of divergence time in years are sensitive to assumptions regarding generation time and mutation rates. Assuming a generation time of 1 year and a mutation rate of 4.1 × 10^−9^, Geraldes et al. [27] estimated the divergence time of *M. m. domesticus* and *M. m. castaneus* as ~ 330 KYA (90% posterior density interval: 220,897 –579,617). For *M. m. domesticus* and *M. m. musculus,* divergence time was estimated as ~ 628 KYA (no CI reported). Divergence time between *M. m. castaneus* and *M. m. musculus* could not be reliably estimated. Given that ML estimates were unreliable and confidence intervals were large, the authors concluded that divergence occurred in the last ~500KYA. Assuming a generation time of 1 year, results from Geraldes et al. [28] suggested a near simultaneous divergence among the three subspecies ~350KYA (*M. m. castaneus* and *M. m. musculus*: 320,764 KYA, no CI reported; *M. m. domesticus* and *M. m. musculus*: 345,752 KYA, no CI reported; *M. m. domesticus* and *M. m. castaneus*: ~ 313,822 KYA, CI: 247,268–372,981). Rescaling our results following assumptions in Geraldes et al. [27] yields broadly consistent divergence time estimates of ~400KYA for *M. m. domesticus* and (*M. m. castaneus, M. m. musculus)* and ~ 320KYA for the subsequent split between *M. m. castaneus, M. m. musculus* (Table S4).

The general agreement among the studies is notable, especially given differences in the number and nature of loci sampled and the geographic range of sampling. Geraldes et al. [27] included just eight loci, including three that were sex-linked and one that was mitochondrial, and sampled intronic regions rather than the intergenic regions sampled in our study. Geraldes et al. [28] surveyed mostly intronic regions of 27 autosomal loci. In addition, while the geographic regions sampled in our study are very similar to those sampled in Geraldes et al. [28], they are a subset of those included in Geraldes et al. [27]. In particular, Geraldes et al. [27] sampled *M. m. castaneus* from China and Taiwan in addition to India. If the additional populations included in Geraldes et al. [27] were more divergent, this would be expected to drive up polymorphism levels and *N*_*e*_ estimates within subspecies. We did not observe this, and our estimates for *N*_*e*_ in *M. m. castaneus* are as high or higher than reported by Geraldes et al. [27]. Moreover, comparisons of population genetic summary statistics suggest that levels of nucleotide variation among the autosomal loci included in the Geraldes et al. [27] study and our random sample of 200 loci are similar (Tables S12, S13 and S14 [45];).

**Fig. 2:**
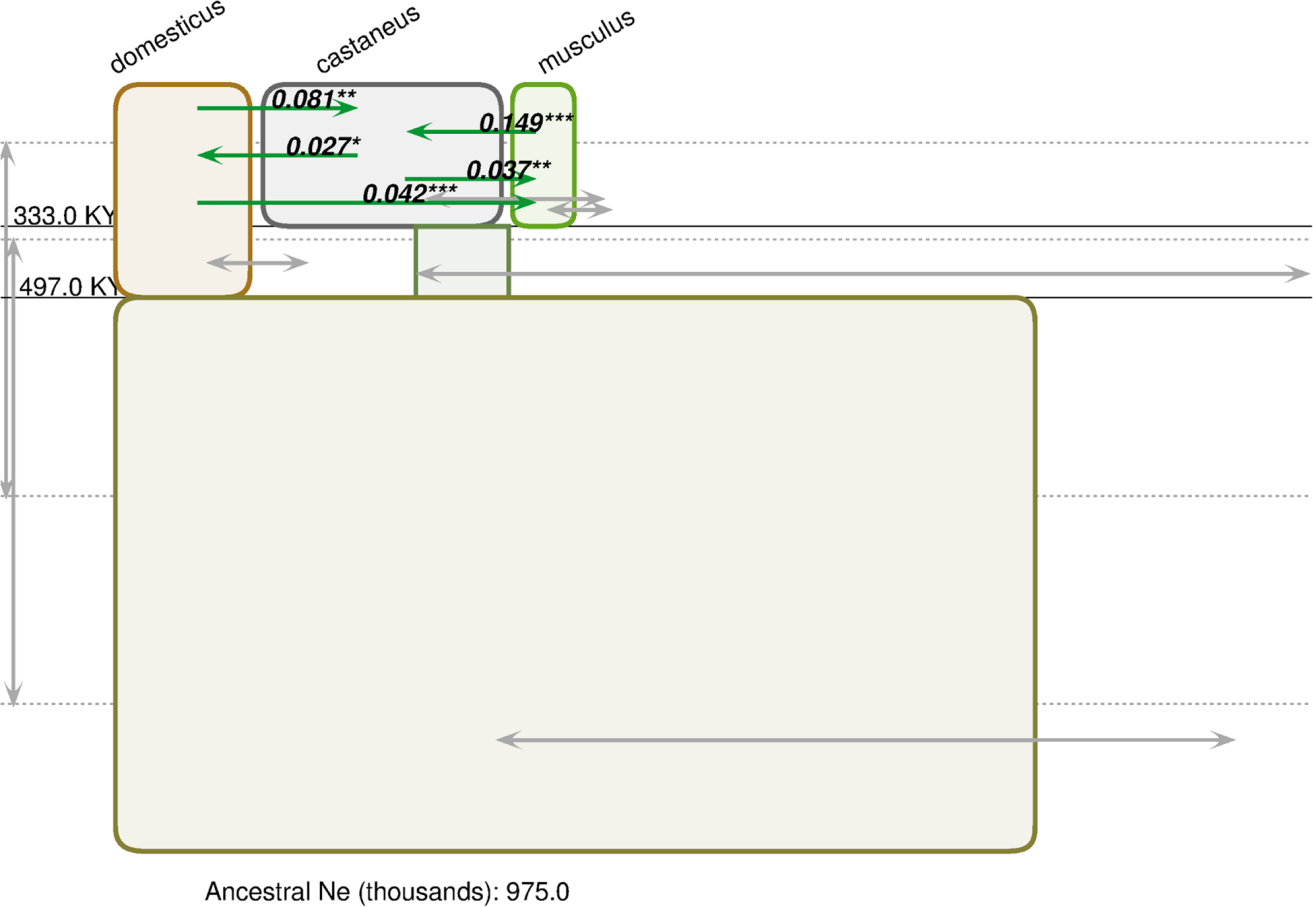
A representation of an estimated IM model generated by IMa3 and the IMfig program [37] for house mice using six nuclear loci from Geraldes et al. [27]. Details are as given in Fig. 1. Estimates assume 1.5 generations/year and 6 × 10^−9^ mutations per base pair per generation

We also reanalyzed data from Geraldes et al. [27] to more directly compare our results to those of previous isolation-with-migration analyses on pairs of species. With a pairwise approach, there was no resolution of the topology. However, our reanalysis including four autosomal and two X-linked loci from [27] supported the (*Mus musculus domesticus,* (*Mus musculus castaneus*, *Mus musculus musculus*)) phylogeny (estimated posterior probability 0.479) compared to the other possible topologies (estimated posterior probabilities 0.315 and 0.206, Table S15). Therefore, while there were differences in some estimates of demographic parameters (e.g. ancestral *N*_*e*_ and divergence times), the overall topology was recovered using IMa3 even with this limited set of loci (Fig. 2, Tables S15, S16, S17 and S18).

## Conclusions

Our joint demographic and phylogenetic analyses refine our understanding of the history of divergence in house mice. Results significantly strengthen evidence for a sister relationship between *M. m. castaneus* and *M. m. musculus* ([25, 35, 36]). The IMa3 analyses also found evidence of gene flow between all pairs of subspecies, but that gene flow into *M. m. domesticus* from *M. m. musculus* was more limited. Estimates of effective population size for the extant subspecies are largely consistent with previous results, with the estimates of *N*_*e*_ in *M. m. castaneus* of on the order of ~ 350,000 -1,100,000, estimates for *M. m. domesticus* of ~ 80,000-240,000, and estimates for *M. m. musculus* of ~ 60,000-170,000. Estimates of divergence time suggest that *M. m. domesticus* split from (*M. m. castaneus* and *M. m. musculus)* in the last 500KYA and potentially as recently as ~130KYA and that the split between *M. m. castaneus* and *M. m. musculus* occurred shortly thereafter, ~ 110-320KYA.

## Methods

### Sequence data

Genomic data were derived from Harr et al. [39]. Briefly, DNA was extracted from samples of mice collected throughout the range of the subspecies in Europe and Asia via either DNeasy kits (Qiagen, Hilden, Germany) or salt extraction [46]. Libraries were then prepared via the TruSeq DNA LT Sample Prep Kit v2 or the Nextera DNA library Prep Kit. Paired-end sequencing of the libraries was performed on either the Hiseq2000 or the NextSeq 500. These reads along with previously published *M. m. castaneus* [29] reads were then mapped to the mm10 genome reference sequence [47] via bwa-mem [48] and a bioinformatics pipeline including Picard (http://broadinstitute.github.io/picard/) and GATK [49] was used to remove duplicates and call variants. Coverage varied among individuals and there was some evidence for relatedness among individuals within subspecies [39]. In addition, there were many more individuals re-sequenced from *M. m. domesticus* and *M. m. musculus* than *M. m. castaneus*. To generate the data file for subsequent analyses, we selected a subset of samples from each subspecies, avoiding individuals with high relatedness scores and/or lower coverage and maximizing geographic representation (S1; *M. m. castaneus*, *n* = 7; *M. m. domesticus*, *n* = 9; *M. m. musculus*, n = 9). Starting from the full population-sorted vcf file (available here, http://wwwuser.gwdg.de/~evolbio/evolgen/wildmouse/vcf), we used GATK to filter out all other individuals and non-variant sites starting with the file including only SNPs that were flagged “PASS” (for filtering details, see [39]).

### IMa3 analyses

Like other genealogy samplers, IMa3 is limited by the assumptions that loci are separated by high recombination, whereas recombination within loci is absent. To generate sampled regions that do not show evidence of recombination, regions were subsampled using the 4-gamete criterion [50]. However, this type of filter does not account for the effect of recombination events that are not detected. To minimize these effects, we sampled non-overlapping regions that passed the 4-gamete criterion, with a minimum number of two SNPs for each sampled region [51] as previously described [52]. Because the genealogies of all the loci must be updated simultaneously in IMa3, when updating the phylogeny [37], runtimes increase greatly when large numbers of loci are used, even when using multiple processors. Given runtime considerations, and from previous experience [37], we focused on data sets of 200 loci. Runs with larger numbers of loci were attempted (e.g. 400 loci), however the Markov-chain simulations showed poor mixing. Sampled regions were selected to exclude: [4] regions within 10,000 base pairs of coding regions, because of possible selective effects [20]; CpG sites, because of the possibility that SNPs in these positions could be caused by more than 1 mutation; and [24] simple repeats, because of possible misalignment within and near repeats. Files were prepared using modifications of scripts available from the PopGen Pipeline Platform [53]. For the 200 sampled loci, the mean locus length was 544 base pairs and the mean number of polymorphic sites was 12.9 (Table S19). Linked selection can create covariation between recombination rate and local estimates of *N*_*e*_. To address this possible bias, we also calculated the recombination rate at each locus using a sex averaged map [54] and the Mouse Map Converter (http://cgd.jax.org/mousemapconverter; Table S19). Recombination rates were calculated by obtaining genetic map positions to either side of the locus (+/− 10,000 bp on either side) and estimating the derivative (rate) by taking the difference in map position and dividing by (20,000 + locus length). We found no significant correlation between recombination rate and variability in our dataset (Figure S2; y=0.0005*x* + 0.0272, R^2^ = 0.0009).

IMa3 runs used uniform prior distributions with upper bounds of 2.0 for population size mutation rate (4*N*μ) parameters, 0.2 for migration rates (*m*/μ) and 1.5 for splitting time (*t*μ). In order to ensure proper mixing of the Markov-chain simulation, a large number (400–480 depending on the run) of Metropolis-coupled [55] chains were used, with 80 or 100 processors for each run. Runs began with a burn-in period of 24 h, followed by 3 or 4 days of sampling. Effective sample sizes were estimated over 1500 phylogenies for all runs. We repeated our analyses following the same protocol with a second set of 200 randomly sampled loci.

For comparison, we also analyzed six loci from Geraldes et al. [27], four on the autosomes and two on the X chromosome. These data were originally analyzed using the older IM program [31] in three pairwise analyses. We aligned the data (A. Geraldes, pers. comm.) for all three species and sampled intervals and assigned mutation rates as given above for the analysis of the 200 locus autosomal data set. Because these loci were longer on average than those used for our main analysis, the uniform prior distributions for the IMa3 run had upper bounds of 10.0 for population size mutation rate (4*N*μ) parameters, 0.2 for migration rates (*m*/μ), and 1.5 for splitting time (*t*μ). A ghost population was not included in the model. A 14-h run (2-h burn-in) using 90 chains and 30 processors yielded results with effective sample sizes above 500.

## Supplementary information

**Additional file 1:Supplementary Table 1.** Collection information and mapping metrics for samples from Harr et al. [39] included in this study. **Supplementary Table 2.** Parameter estimates with 95% confidence limits for the most probable model of three subspecies without a ghost population. **Supplementary Table 3.** Parameter estimates with 95% confidence limits for the most probable model of three subspecies with a ghost population. **Supplementary Table 4.** Estimated split times (years) with 95% confidence limits. **Supplementary Table 5.** Estimated effective population sizes (*N*_*e*_) with 95% confidence limits (no ghost population). **Supplementary Table 6.** Estimated effective population sizes (*N*_*e*_) with 95% confidence limits (with a ghost population). **Supplementary Table 7.** Estimated population migration rates with 95% confidence limits for the most probable topology with and without a ghost population included. **Supplementary Table 8.** Phylogeny results from the analysis of a second set of 200 random autosomal loci without a ghost population (compare with values from Table 1). **Supplementary Table 9.** Parameter estimates with 95% confidence limits for the most probable model of three subspecies without a ghost population for a second set of 200 random autosomal loci. **Supplementary Table 10.** Estimated split times (years) and effective population sizes (*N*_*e*_) with 95% confidence limits using a second set of 200 random autosomal loci (without a ghost population). **Supplementary Table 11.** Estimated population migration rates with 95% confidence limits for the most probable topology using a second set of 200 random autosomal loci (without a ghost population). **Supplementary Table 12.** ${\hat{\theta}}_{\pi }$ / ${\hat{D}}_{xy}$ matrix for the 200 randomly selected autosomal loci from the genomic data. **Supplementary Table 13.** ${\hat{\theta}}_{\pi }$ / ${\hat{D}}_{xy}$ matrix for the 4 autosomal loci from Geraldes et al. [27]. **Supplementary Table 14.** *F*_*st*_ between subspecies was calculated as the mean across loci. Locus specific values of *F*_*st*_ were calculated using pairwise differences between sequences following Hudson, Slatkin, and Maddison [45]. **Supplementary Table 15.** Phylogeny results from the re-analysis of four autosomal and two X-linked loci included in Geraldes et al., [27]. **Supplementary Table 16.** Parameter estimates with 95% confidence limits for the most probable model of three subspecies using four autosomal and two X-linked loci included in Geraldes et al. [27]. **Supplementary Table 17.** Estimated split times (years) and effective population sizes (*N*_*e*_) with 95% confidence limits for an analysis using four autosomal and two X-linked loci included in Geraldes et al. [27]. **Supplementary Table 18.** Estimated population migration rates with 95% confidence limits given the most probable topology for an analysis using four autosomal and two X-linked loci included in Geraldes et al. [27]. **Supplementary Table 19.** Summary information for 200 loci, including location on the chromosome, length, number of variable sites, and recombination rate (cM/Mb). Recombination rates were based on sex-averaged maps [54] as reported at http://cgd.jax.org/mousemapconverter/. **Supplementary Figure 1.** A representation of an estimated Isolation with Migration model generated by IMa3 and the IMfig program [37] for house mice using a set of 200 alternative random autosomal loci. Details are as given in Fig. 1. Estimates assume 0.75 generations/year and 6 × 10^−9^ mutations per base pair per generation. **Supplementary Figure 2.** SNP density plotted against recombination rate for 200 sampled loci for **A)** all loci (*y* = 0.0005*x* + 0.0272, R^2^ = 0.0009) and **B)** all loci excluding outliers with recombination rate greater than 2 cM/Mb (*y* = 0.0008*x* + 0.271, R^2^ = 0.0007; data from Table S19).

## Data Availability

All data included in this study was published in [38] and is available from: http://wwwuser.gwdg.de/~evolbio/evolgen/wildmouse/vcf/AllMouse.vcf_90_recalibrated_snps_raw_indels_reheader_PopSorted.PASS.vcf.gz. Input files, output files, and commands for the IMa3 analysis of the random sample of 200 loci and for the re-analysis of the nuclear loci from Geraldes et al. [27] have been deposited in Dryad (accessible under: doi:10.5061/dryad.qrfj6q5cp).

## Abbreviations

bp: Basepair
CI: Confidence interval
IM: Isolation with migration
MCMC: Markov chain Monte Carlo
*N*_*e*_: Effective population size
