# Supplementary material for: Further resolution of the house mouse (Mus musculus) phylogeny by integration over isolation-with-migration histories
Source: BMC Evol Biol. 2020 Sep 15;20:120. doi: 10.1186/s12862-020-01666-9 (PMC7493149; doi:10.1186/s12862-020-01666-9)
Supplement: Supplementary file 1 — Additional file 1:Supplementary Table 1. Collection information and mapping metrics for samples from Harr et al. [39] included in this study. Supplementary Table 2. Parameter estimates with 95% confidence limits for the most probable model of three subspecies without a ghost population. Supplementary Table 3. Parameter estimates with 95% confidence limits for the most probable model of three subspecies with a ghost population. Supplementary Table 4. Estimated split times (years) with 95% confidence limits. Supplementary Table 5. Estimated effective population sizes (Ne) with 95% confidence limits (no ghost population). Supplementary Table 6. Estimated effective population sizes (Ne) with 95% confidence limits (with a ghost population). Supplementary Table 7. Estimated population migration rates with 95% confidence limits for the most probable topology with and without a ghost population included. Supplementary Table 8. Phylogeny results from the analysis of a second set of 200 random autosomal loci without a ghost population (compare with values from Table 1). Supplementary Table 9. Parameter estimates with 95% confidence limits for the most probable model of three subspecies without a ghost population for a second set of 200 random autosomal loci. Supplementary Table 10. Estimated split times (years) and effective population sizes (Ne) with 95% confidence limits using a second set of 200 random autosomal loci (without a ghost population). Supplementary Table 11. Estimated population migration rates with 95% confidence limits for the most probable topology using a second set of 200 random autosomal loci (without a ghost population). Supplementary Table 12. ${\hat{\theta}}_{\pi }$θ^π / [file 12862_2020_1666_MOESM1_ESM.docx]

Supplementary Table 1. Collection information and mapping metrics for samples from Harr *et al.* (2016) included in this study.

| Subspecies | Sample ID | Location | Field Sex | Total Reads (x10^8^) | High quality mapped (%) | Autosome fold coverage | ENA study | ENA sample |
| --- | --- | --- | --- | --- | --- | --- | --- | --- |
| *M. m. castaneus* | H36 | India (Himalaya) - 8 | female | 6.38 | 90 | 19 | PRJEB2176 | ERS003050 |
| *M. m. castaneus* | H34 | India (Himalaya) - 8 | male | 6.63 | 91.7 | 21 | PRJEB2176 | ERS003049 |
| *M. m. castaneus* | H30 | India (Himalaya) - 8 | female | 7.03 | 87.3 | 21 | PRJEB2176 | ERS003044 |
| *M. m. castaneus* | H28 | India (Himalaya) - 8 | male | 4.82 | 90.6 | 15 | PRJEB2176 | ERS003048 |
| *M. m. castaneus* | H26 | India (Himalaya) - 8 | female | 5.67 | 87.7 | 17 | PRJEB2176 | ERS003046 |
| *M. m. castaneus* | H14 | India (Himalaya) - 8 | female | 5.66 | 88.5 | 17 | PRJEB2176 | ERS003041 |
| *M. m. castaneus* | H12 | India (Himalaya) - 8 | male | 6.12 | 91.2 | 20 | PRJEB2176 | ERS003051 |
| *M. m. domesticus* | F1B | France (Massif Central) - 1 | male | 6.93 | 95.7 | 23 | PRJEB9450 | ERS739382 |
| *M. m. domesticus* | C1 | France (Massif Central) - 1 | male | 6.18 | 94.3 | 20 | PRJEB9450 | ERS739384 |
| *M. m. domesticus* | 18B | France (Massif Central) - 1 | male | 7.3 | 96.1 | 24 | PRJEB9450 | ERS739381 |
| *M. m. domesticus* | TP3-92 | Germany (Cologne-Bonn) - 2 | male | 7.04 | 95.1 | 23 | PRJEB9450 | ERS739374 |
| *M. m. domesticus* | TP17-2 | Germany (Cologne-Bonn) - 2 | male | 7.01 | 96 | 24 | PRJEB9450 | ERS739376 |
| *M. m. domesticus* | TP1 | Germany (Cologne-Bonn) - 2 | male | 6.84 | 94.5 | 23 | PRJEB9450 | ERS739373 |
| *M. m. domesticus* | JR7-F1C | Iran (Ahvaz) - 4 | male | 6.06 | 92.2 | 18 | PRJEB9450 | ERS739391 |
| *M. m. domesticus* | JR5-F1C | Iran (Ahvaz) - 4 | male | 5.79 | 90.6 | 17 | PRJEB9450 | ERS739390 |
| *M. m. domesticus* | AH23 | Iran (Ahvaz) - 4 | male | 8.07 | 89.6 | 24 | PRJEB9450 | ERS739395 |
| *M. m. musculus* | 444 | Afghanistan (Mazar-e-Sharif) - 7 | male | 11.46 | 94.62 | 18 | PRJEB14167 | ERS1180813 |
| *M. m. musculus* | 435 | Afghanistan (Mazar-e-Sharif) - 7 | female | 12.17 | 96.65 | 19 | PRJEB14167 | ERS1180812 |
| *M. m. musculus* | 413 | Afghanistan (Mazar-e-Sharif) - 7 | male | 11.96 | 96.05 | 21 | PRJEB14167 | ERS1180809 |

Supplementary Table 2. Parameter estimates with 95% confidence limits for the most probable model of three subspecies without a ghost population.

| Parameter | Mutation Rate |  | MLE | 95%Lo | 95%Hi |
| --- | --- | --- | --- | --- | --- |
| *tµ* CAS, MUS | - |  | 0.3668 | 0.3202 | 0.4373 |
| *tµ* DOM, (CAS, MUS) | - |  | 0.4808 | 0.4238 | 0.5467 |
| *4Nµ* CAS | - |  | 3.357 | 2.987 | 3.833 |
| *4Nµ* DOM | - |  | 0.7325 | 0.6575 | 0.8175 |
| *4Nµ* MUS | - |  | 0.5175 | 0.4575 | 0.5825 |
| *4Nµ* (CAS, MUS) | - |  | 1.308 | 0.6525 | 2.268 |
| *4Nµ* (CAS, MUS, DOM) | - |  | 0.9425 | 0.7975 | 1.103 |
| *m/µ* CAS>DOM | 6x10^-9^ |  | 0.1245^***^ | 0.0593 | 0.1909 |
| *m/µ* DOM>CAS | 6x10^-9^ |  | 0.1999^***^ | 0.0931 | 0.1987 |
| *m/µ* CAS>MUS | 6x10^-9^ |  | 0.1999^***^ | 0.1007 | 0.1991 |
| *m/µ* MUS>CAS | 6x10^-9^ |  | 0.0991^*^ | 0.0189 | 0.1895 |
| *m/µ* DOM>MUS | 6x10^-9^ |  | 0.0127 | 0.0015 | 0.0969 |
| *m/µ* MUS>DOM | 6x10^-9^ |  | 0.1999^***^ | 0.1347 | 0.1993 |
| *m/µ* DOM>(CAS, MUS) | 6x10^-9^ |  | 0.1999 | 0.0053 | 0.1951 |
| *m/µ* (CAS, MUS)>DOM | 6x10^-9^ |  | 0.1999 | 0.0059 | 0.1959 |
| *m/µ* CAS>DOM | 4.1x10^-9^ |  | 0.0851^***^ | 0.0405 | 0.1304 |
| *m/µ* DOM>CAS | 4.1x10^-9^ |  | 0.1366^***^ | 0.0636 | 0.1358 |
| *m/µ* CAS>MUS | 4.1x10^-9^ |  | 0.1366^***^ | 0.0688 | 0.1361 |
| *m/µ* MUS>CAS | 4.1x10^-9^ |  | 0.0677^*^ | 0.0129 | 0.1295 |
| *m/µ* DOM>MUS | 4.1x10^-9^ |  | 0.0087 | 0.0010 | 0.0662 |
| *m/µ* MUS>DOM | 4.1x10^-9^ |  | 0.1366^***^ | 0.0920 | 0.1362 |
| *m/µ* DOM>(CAS, MUS) | 4.1x10^-9^ |  | 0.1366 | 0.0036 | 0.1333 |
| *m/µ* (CAS, MUS)>DOM | 4.1x10^-9^ |  | 0.1366 | 0.0040 | 0.1339 |

Subspecies names are abbreviated, CAS for *Mus musculus castaneus*, DOM for *Mus musculus domesticus,* and MUS for *Mus musculus musculus.* For each parameter, the estimated value is the maximum of the estimated marginal posterior probability density for that parameter. Population size and splitting time parameters are shown scaled by *µ*, and migration rate parameters are shown scaled by the inverse of *µ*, where *µ* is the geometric mean of the mutation rates (Hey & Nielsen 2004). The direction of gene flow is indicated by “>”, with gene flow from the subspecies/group listed second into the subspecies listed first (^*^p < 0.05, ^**^p< 0.01, ^***^p < 0.001).

Supplementary Table 3. Parameter estimates with 95% confidence limits for the most probable model of three subspecies with a ghost population.

| Parameter | Mutation Rate | MLE | 95%Lo | 95%Hi |
| --- | --- | --- | --- | --- |
| *tµ* CAS, MUS | - | 0.3698 | 0.3172 | 0.4253 |
| *tµ* DOM, (CAS, MUS) | - | 0.4477 | 0.3937 | 0.5092 |
| *tµ* ANC | - | 0.8558 | 0.6532 | 1.217 |
| *4Nµ* CAS | - | 3.188 | 2.812 | 3.678 |
| *4Nµ* DOM | - | 0.6925 | 0.6125 | 0.7775 |
| *4Nµ* MUS | - | 0.5125 | 0.4525 | 0.5775 |
| *4Nµ* ghost | - | 0.8025 | 0.2325 | 4.798 |
| *4Nµ* (CAS, MUS) | - | 0.8475 | 0.3175 | 1.688 |
| *4Nµ* (CAS, MUS, DOM) | - | 0.9325 | 0.7075 | 1.397 |
| *4Nµ* ANC | - | 0.7075 | 0.2775 | 0.9625 |
| *m/µ* CAS>DOM | 6x10^-9^ | 0.1175^***^ | 0.0525 | 0.1901 |
| *m/µ* DOM>CAS | 6x10^-9^ | 0.1799^***^ | 0.0609 | 0.1975 |
| *m/µ* CAS>MUS | 6x10^-9^ | 0.1999^***^ | 0.0833 | 0.1987 |
| *m/µ* MUS>CAS | 6x10^-9^ | 0.0805^*^ | 0.0099 | 0.1839 |
| *m/µ* DOM>MUS | 6x10^-9^ | 0.0063 | 0.0011 | 0.0851 |
| *m/µ* MUS>DOM | 6x10^-9^ | 0.1999^***^ | 0.1339 | 0.1993 |
| *m/µ* DOM>(CAS, MUS) | 6x10^-9^ | 0.0411 | 0.0051 | 0.1949 |
| *m/µ* (CAS, MUS)>DOM | 6x10^-9^ | 0.1999 | 0.0055 | 0.1953 |
| *m/µ* CAS>DOM | 4.1x10^-9^ | 0.0803^***^ | 0.0359 | 0.1299 |
| *m/µ* DOM>CAS | 4.1x10^-9^ | 0.1229^***^ | 0.0416 | 0.1350 |
| *m/µ* CAS>MUS | 4.1x10^-9^ | 0.1366^***^ | 0.0569 | 0.1358 |
| *m/µ* MUS>CAS | 4.1x10^-9^ | 0.0550^*^ | 0.0068 | 0.1257 |
| *m/µ* DOM>MUS | 4.1x10^-9^ | 0.0043 | 0.0008 | 0.0582 |
| *m/µ* MUS>DOM | 4.1x10^-9^ | 0.1366^***^ | 0.0915 | 0.1362 |
| *m/µ* DOM>(CAS, MUS) | 4.1x10^-9^ | 0.0281 | 0.0035 | 0.1332 |
| *m/µ* (CAS, MUS)>DOM | 4.1x10^-9^ | 0.1366 | 0.0038 | 0.1335 |

Subspecies names are abbreviated, CAS for *Mus musculus castaneus*, DOM for *Mus musculus domesticus,* and MUS for *Mus musculus musculus.* For each parameter, the estimated value is the maximum of the estimated marginal posterior probability density for that parameter. Population size and splitting time parameters are shown scaled by *µ*, and migration rate parameters are shown scaled by the inverse of *µ*, where *µ* is the geometric mean of the mutation rates (Hey & Nielsen 2004). The direction of gene flow is indicated by “>”, with gene flow from the subspecies/group listed second into the subspecies listed first (^*^p < 0.05, ^**^p< 0.01, ^***^p < 0.001).

Supplementary Table 4. Estimated split times (years) with 95% confidence limits.

| Model | Mutation Rate | Generations per year | Parameter | MLE | 95%Lo | 95%Hi |
| --- | --- | --- | --- | --- | --- | --- |
| Without ghost | 6 x 10^-9^ | 1 | Time since split between CAS and MUS | 217,833 | 190,215 | 259,708 |
|  | 6 x 10^-9^ | 1 | Time since split between DOM and (CAS, MUS) | 285,544 | 251,689 | 324,745 |
|  | 6 x 10^-9^ | 1.5 | Time since split between CAS and MUS | 163,375 | 142,661 | 194,781 |
|  | 6 x 10^-9^ | 1.5 | Time since split between DOM and (CAS, MUS) | 214,158 | 188,767 | 243,559 |
|  | 6 x 10^-9^ | 2 | Time since split between CAS and MUS | 108,917 | 95,107 | 129,854 |
|  | 6 x 10^-9^ | 2 | Time since split between DOM and (CAS, MUS) | 142,772 | 125,845 | 162,373 |
|  | 4.1 x 10^-9^ | 1 | Time since split between CAS and MUS | 318,780 | 278,363 | 380,060 |
|  | 4.1 x 10^-9^ | 1 | Time since split between DOM and (CAS, MUS) | 417,869 | 368,326 | 475,237 |
|  | 4.1 x 10^-9^ | 1.5 | Time since split between CAS and MUS | 239,085 | 208,772 | 285,045 |
|  | 4.1 x 10^-9^ | 1.5 | Time since split between DOM and (CAS, MUS) | 313,402 | 276,244 | 356,428 |
|  | 4.1 x 10^-9^ | 2 | Time since split between CAS and MUS | 159,390 | 139,181 | 190,030 |
|  | 4.1 x 10^-9^ | 2 | Time since split between DOM and (CAS, MUS) | 208,935 | 184,163 | 237,619 |
| With ghost | 6 x 10^-9^ | 1 | Time since split between CAS and MUS | 220,223 | 188,955 | 253,279 |
|  | 6 x 10^-9^ | 1 | Time since split between DOM and (CAS, MUS) | 266,680 | 234,517 | 303,309 |
|  | 6 x 10^-9^ | 1.5 | Time since split between CAS and MUS | 165,167 | 141,716 | 189,959 |
|  | 6 x 10^-9^ | 1.5 | Time since split between DOM and (CAS, MUS) | 200,010 | 175,888 | 227,482 |
|  | 6 x 10^-9^ | 2 | Time since split between CAS and MUS | 110,111 | 94,477 | 126,639 |
|  | 6 x 10^-9^ | 2 | Time since split between DOM and (CAS, MUS) | 133,340 | 117,259 | 151,655 |
|  | 4.1 x 10^-9^ | 1 | Time since split between CAS and MUS | 322,277 | 276,519 | 370,652 |
|  | 4.1 x 10^-9^ | 1 | Time since split between DOM and (CAS, MUS) | 390,263 | 343,196 | 443,867 |
|  | 4.1 x 10^-9^ | 1.5 | Time since split between CAS and MUS | 241,708 | 207,389 | 277,989 |
|  | 4.1 x 10^-9^ | 1.5 | Time since split between DOM and (CAS, MUS) | 292,698 | 257,397 | 332,900 |
|  | 4.1 x 10^-9^ | 2 | Time since split between CAS and MUS | 161,139 | 138,260 | 185,326 |
|  | 4.1 x 10^-9^ | 2 | Time since split between DOM and (CAS, MUS) | 195,132 | 171,598 | 221,934 |

| Supplementary Table 5. Estimated effective population sizes (*N_e_*) with 95% confidence limits (no ghost population). | | | | | |
| --- | --- | --- | --- | --- | --- |
| Mutation Rate | Generations/year | Parameter | MLE | 95%Lo | 95%Hi |
| 6 x 10^-9^ | 1 | *N_e_* CAS | 373,914 | 332,708 | 426,813 |
| 6 x 10^-9^ | 1 | *N_e_* DOM | 81,576 | 73,224 | 91,043 |
| 6 x 10^-9^ | 1 | *N_e_* MUS | 57,632 | 50,951 | 64,871 |
| 6 x 10^-9^ | 1 | *N_e_* Ancestral population (CAS, MUS) | 145,612 | 72,667 | 252,524 |
| 6 x 10^-9^ | 1 | *N_e_* Ancestral population (CAS, MUS, DOM) | 104,963 | 88,815 | 122,782 |
| 6 x 10^-9^ | 1.5 | *N_e_* CAS | 498,552 | 443,611 | 569,084 |
| 6 x 10^-9^ | 1.5 | *N_e_* DOM | 108,768 | 97,632 | 121,390 |
| 6 x 10^-9^ | 1.5 | *N_e_* MUS | 76,843 | 67,934 | 86,495 |
| 6 x 10^-9^ | 1.5 | *N_e_* Ancestral population (CAS, MUS) | 194,149 | 96,889 | 336,699 |
| 6 x 10^-9^ | 1.5 | *N_e_* Ancestral population (CAS, MUS, DOM) | 139,951 | 118,420 | 163,709 |
| 6 x 10^-9^ | 2 | *N_e_* CAS | 747,828 | 665,417 | 853,626 |
| 6 x 10^-9^ | 2 | *N_e_* DOM | 163,152 | 146,448 | 182,085 |
| 6 x 10^-9^ | 2 | *N_e_* MUS | 115,265 | 101,901 | 129,743 |
| 6 x 10^-9^ | 2 | *N_e_* Ancestral population (CAS, MUS) | 291,224 | 145,334 | 505,049 |
| 6 x 10^-9^ | 2 | *N_e_* Ancestral population (CAS, MUS, DOM) | 209,927 | 177,630 | 245,564 |
| 4.1 x 10^-9^ | 1 | *N_e_* CAS | 547,191 | 486,890 | 624,604 |
| 4.1 x 10^-9^ | 1 | *N_e_* DOM | 119,380 | 107,157 | 133,233 |
| 4.1 x 10^-9^ | 1 | *N_e_* MUS | 84,340 | 74,562 | 94,934 |
| 4.1 x 10^-9^ | 1 | *N_e_* Ancestral population (CAS, MUS) | 213,090 | 106,342 | 369,548 |
| 4.1 x 10^-9^ | 1 | *N_e_* Ancestral population (CAS, MUS, DOM) | 153,605 | 129,973 | 179,681 |
| 4.1 x 10^-9^ | 1.5 | *N_e_* CAS | 729,588 | 649,187 | 832,806 |
| 4.1 x 10^-9^ | 1.5 | *N_e_* DOM | 159,173 | 142,876 | 177,644 |
| 4.1 x 10^-9^ | 1.5 | *N_e_* MUS | 112,453 | 99,416 | 126,578 |
| 4.1 x 10^-9^ | 1.5 | *N_e_* Ancestral population (CAS, MUS) | 284,120 | 141,789 | 492,730 |
| 4.1 x 10^-9^ | 1.5 | *N_e_* Ancestral population (CAS, MUS, DOM) | 204,806 | 173,298 | 239,574 |
| 4.1 x 10^-9^ | 2 | *N_e_* CAS | 1,094,382 | 973,780 | 1,249,209 |
| 4.1 x 10^-9^ | 2 | *N_e_* DOM | 238,759 | 214,314 | 266,466 |
| 4.1 x 10^-9^ | 2 | *N_e_* MUS | 168,680 | 149,123 | 189,867 |
| 4.1 x 10^-9^ | 2 | *N_e_* Ancestral population (CAS, MUS) | 426,181 | 212,683 | 739,095 |
| 4.1 x 10^-9^ | 2 | *N_e_* Ancestral population (CAS, MUS, DOM) | 307,210 | 259,946 | 359,361 |

| Supplementary Table 6. Estimated effective population sizes (*N_e_*) with 95% confidence limits (with a ghost population). | | | | | |
| --- | --- | --- | --- | --- | --- |
| Mutation Rate | Generations/year | Parameter | MLE | 95%Lo | 95%Hi |
| 6 x 10^-9^ | 1 | *N_e_* CAS | 355,964 | 314,086 | 410,685 |
| 6 x 10^-9^ | 1 | *N_e_* DOM | 76,777 | 68,401 | 86,828 |
| 6 x 10^-9^ | 1 | *N_e_* MUS | 57,233 | 50,533 | 64,493 |
| 6 x 10^-9^ | 1 | *N_e_* Ancestral population (CAS, MUS) | 94,645 | 35,457 | 188,452 |
| 6 x 10^-9^ | 1 | *N_e_* Ancestral population (CAS, MUS, DOM) | 104,137 | 79,010 | 156,066 |
| 6 x 10^-9^ | 1.5 | *N_e_* CAS | 474,619 | 418,781 | 547,580 |
| 6 x 10^-9^ | 1.5 | *N_e_* DOM | 102,369 | 91,201 | 115,770 |
| 6 x 10^-9^ | 1.5 | *N_e_* MUS | 76,311 | 67,377 | 85,990 |
| 6 x 10^-9^ | 1.5 | *N_e_* Ancestral population (CAS, MUS) | 126,193 | 47,276 | 251,269 |
| 6 x 10^-9^ | 1.5 | *N_e_* Ancestral population (CAS, MUS, DOM) | 138,849 | 105,347 | 208,088 |
| 6 x 10^-9^ | 2 | *N_e_* CAS | 711,929 | 628,172 | 821,370 |
| 6 x 10^-9^ | 2 | *N_e_* DOM | 153,554 | 136,802 | 173,655 |
| 6 x 10^-9^ | 2 | *N_e_* MUS | 114,467 | 101,066 | 128,985 |
| 6 x 10^-9^ | 2 | *N_e_* Ancestral population (CAS, MUS) | 189,290 | 70,914 | 376,904 |
| 6 x 10^-9^ | 2 | *N_e_* Ancestral population (CAS, MUS, DOM) | 208,274 | 158,021 | 312,132 |
| 4.1 x 10^-9^ | 1 | *N_e_* CAS | 520,923 | 459,638 | 601,002 |
| 4.1 x 10^-9^ | 1 | *N_e_* DOM | 112,356 | 100,099 | 127,065 |
| 4.1 x 10^-9^ | 1 | *N_e_* MUS | 83,756 | 73,950 | 94,379 |
| 4.1 x 10^-9^ | 1 | *N_e_* Ancestral population (CAS, MUS) | 138,505 | 51,888 | 275,783 |
| 4.1 x 10^-9^ | 1 | *N_e_* Ancestral population (CAS, MUS, DOM) | 152,395 | 115,625 | 228,389 |
| 4.1 x 10^-9^ | 1.5 | *N_e_* CAS | 694,564 | 612,850 | 801,337 |
| 4.1 x 10^-9^ | 1.5 | *N_e_* DOM | 149,808 | 133,465 | 169,420 |
| 4.1 x 10^-9^ | 1.5 | *N_e_* MUS | 111,675 | 98,600 | 125,839 |
| 4.1 x 10^-9^ | 1.5 | *N_e_* Ancestral population (CAS, MUS) | 184,673 | 69,184 | 367,711 |
| 4.1 x 10^-9^ | 1.5 | *N_e_* Ancestral population (CAS, MUS, DOM) | 203,194 | 154,166 | 304,519 |
| 4.1 x 10^-9^ | 2 | *N_e_* CAS | 1,041,847 | 919,275 | 1,202,005 |
| 4.1 x 10^-9^ | 2 | *N_e_* DOM | 224,712 | 200,197 | 254,129 |
| 4.1 x 10^-9^ | 2 | *N_e_* MUS | 167,512 | 147,901 | 188,759 |
| 4.1 x 10^-9^ | 2 | *N_e_* Ancestral population (CAS, MUS) | 277,009 | 103,777 | 551,566 |
| 4.1 x 10^-9^ | 2 | *N_e_* Ancestral population (CAS, MUS, DOM) | 304,790 | 231,250 | 456,779 |

Supplementary Table 7. Estimated population migration rates with 95% confidence limits for the most probable topology with and without a ghost population included.

| Model | Value | MLE | 95%Lo | 95%Hi |
| --- | --- | --- | --- | --- |
| Without ghost | 2*Nm* CAS>DOM | 0.2103 | 0.1014 | 0.3228 |
|  | 2*Nm* CAS>MUS | 0.3078 | 0.1727 | 0.3564 |
|  | 2*Nm* DOM>CAS | 0.0658 | 0.0348 | 0.0754 |
|  | 2*Nm* DOM>MUS | 0.0051 | 0.0006 | 0.0350 |
|  | 2*Nm* DOM>(CAS, MUS) | 0.0615 | 0.0019 | 0.0728 |
|  | 2*Nm* MUS>CAS | 0.0261 | 0.0050 | 0.0481 |
|  | 2*Nm* MUS>DOM | 0.0476 | 0.0347 | 0.0548 |
|  | 2*Nm* (CAS, MUS)>DOM | 0.0512 | 0.0037 | 0.1672 |
| With ghost | 2*Nm* CAS>DOM | 0.1888 | 0.0860 | 0.3053 |
|  | 2*Nm* CAS>MUS | 0.2881 | 0.1365 | 0.3384 |
|  | 2*Nm* CAS>G | 0.2821 | 0.0701 | 0.3310 |
|  | 2*Nm* DOM>CAS | 0.0591 | 0.0213 | 0.0701 |
|  | 2*Nm* DOM>MUS | 0.0022 | 0.0004 | 0.0291 |
|  | 2*Nm* DOM>G | 0.0277 | 0.0051 | 0.0608 |
|  | 2*Nm* DOM>(CAS, MUS) | 0.0155 | 0.0018 | 0.0686 |
|  | 2*Nm* MUS>CAS | 0.0209 | 0.0026 | 0.0462 |
|  | 2*Nm* MUS>DOM | 0.0472 | 0.0343 | 0.0545 |
|  | 2*Nm* MUS>G | 0.0021 | 0.0003 | 0.0232 |
|  | 2*Nm* G>CAS | 0.0017 | 0.0027 | 0.3686 |
|  | 2*Nm* G>DOM | 0.0002 | 0.0027 | 0.3676 |
|  | 2*Nm* G>MUS | 0.0017 | 0.0022 | 0.3671 |
|  | 2*Nm* G>(CAS, MUS) | 0.0012 | 0.0022 | 0.3671 |
|  | 2*Nm* G>(CAS, MUS, DOM) | 0.0017 | 0.0027 | 0.3721 |
|  | 2*Nm* (CAS, MUS)>DOM | 0.0062 | 0.0022 | 0.1202 |
|  | 2*Nm* (CAS, MUS)>G | 0.0062 | 0.0017 | 0.1177 |
|  | 2*Nm* (CAS, MUS, DOM)>G | 0.0002 | 0.0022 | 0.1047 |

Subspecies names are abbreviated, CAS for *Mus musculus castaneus*, DOM for *Mus musculus domesticus,* MUS for *Mus musculus musculus,* and G for a ghost population*.* The direction of gene flow is indicated by “>”, with gene flow from the subspecies/group listed second into the subspecies listed first.

Supplementary Table 8. Phylogeny results from the analysis of a second set of 200 random autosomal loci without a ghost population (compare with values from Table 1).

| Species Tree | Count | Frequency |
| --- | --- | --- |
| (1,(0,2)3)4 | 81,155 | 0.7780 |
| (2,(0,1)3)4 | 16,276 | 0.1560 |
| (0,(1,2)3)4 | 6,878 | 0.0659 |

The three subspecies are numbered from 0 to 2 (corresponding to *M. m. castaneus*, *M. m. domesticus*, and *M. m. musculus,* respectively). Ancestral populations are numbered beginning with 3, and are ordered numerically in time (i.e., 4 is the ancestor of all populations).

Supplementary Table 9. Parameter estimates with 95% confidence limits for the most probable model of three subspecies without a ghost population for a second set of 200 random autosomal loci.

| Parameter | Mutation Rate | MLE | 95%Lo | 95%Hi |
| --- | --- | --- | --- | --- |
| *tµ* CAS, MUS | - | 0.343 | 0.295 | 0.436 |
| *tµ* DOM, (CAS, MUS) | - | 0.457 | 0.404 | 0.515 |
| *4Nµ* CAS | - | 3.553 | 3.147 | 4.117 |
| *4Nµ* DOM | - | 0.708 | 0.633 | 0.798 |
| *4Nµ* MUS | - | 0.418 | 0.368 | 0.478 |
| *4Nµ* (CAS, MUS) | - | 1.708 | 0.978 | 4.593 |
| *4Nµ* (CAS, MUS, DOM) | - | 0.653 | 0.533 | 0.793 |
| *m/µ* CAS>DOM | 6x10^-9^ | 0.127^***^ | 0.049 | 0.193 |
| *m/µ* DOM>CAS | 6x10^-9^ | 0.090^*^ | 0.017 | 0.189 |
| *m/µ* CAS>MUS | 6x10^-9^ | 0.200^***^ | 0.081 | 0.199 |
| *m/µ* MUS>CAS | 6x10^-9^ | 0.182^**^ | 0.044 | 0.197 |
| *m/µ* DOM>MUS | 6x10^-9^ | 0.173^***^ | 0.098 | 0.198 |
| *m/µ* MUS>DOM | 6x10^-9^ | 0.200^***^ | 0.107 | 0.199 |
| *m/µ* DOM>(CAS, MUS) | 6x10^-9^ | 0.118 | 0.005 | 0.195 |
| *m/µ* (CAS, MUS)>DOM | 6x10^-9^ | 0.200 | 0.006 | 0.196 |

Subspecies names are abbreviated, CAS for *Mus musculus castaneus*, DOM for *Mus musculus domesticus,* and MUS for *Mus musculus musculus.* For each parameter, the estimated value is the maximum of the estimated marginal posterior probability density for that parameter. Population size and splitting time parameters are shown scaled by *µ*, and migration rate parameters are shown scaled by the inverse of *µ*, where *µ* is the geometric mean of the mutation rates (Hey & Nielsen 2004). The direction of gene flow is indicated by “>”, with gene flow from the subspecies/group listed second into the subspecies listed first (^*^p < 0.05, ^**^p< 0.01, ^***^p < 0.001).

Supplementary Table 10. Estimated split times (years) and effective population sizes (*N_e_*) with 95% confidence limits using a second set of 200 random autosomal loci (without a ghost population).

| Parameter | MLE | 95%Lo | 95%Hi |
| --- | --- | --- | --- |
| Time since split between CAS and MUS | 139,131 | 119,647 | 176,882 |
| Time since split between DOM and (CAS, MUS) | 185,407 | 164,096 | 209,153 |
| *N_e_* CAS | 480,684 | 425,884 | 557,133 |
| *N_e_* DOM | 95,731 | 85,583 | 107,909 |
| *N_e_* MUS | 56,491 | 49,726 | 64,610 |
| *N_e_* Ancestral population (CAS, MUS) | 231,039 | 132,264 | 621,405 |
| *N_e_* Ancestral population (CAS, MUS, DOM) | 88,289 | 72,052 | 107,232 |

Estimates assume 1.5 generations/year and $6\times{10}^{-9}$ mutations per base pair per generation.

Supplementary Table 11. Estimated population migration rates with 95% confidence limits for the most probable topology using a second set of 200 random autosomal loci (without a ghost population)*.*

| Value | MLE | 95%Lo | 95%Hi |
| --- | --- | --- | --- |
| 2*Nm* CAS>DOM | 0.226 | 0.089 | 0.352 |
| 2*Nm* CAS>MUS | 0.317 | 0.148 | 0.375 |
| 2*Nm* DOM>CAS | 0.034 | 0.006 | 0.065 |
| 2*Nm* DOM>MUS | 0.061 | 0.035 | 0.073 |
| 2*Nm* MUS>CAS | 0.035 | 0.009 | 0.042 |
| 2*Nm* MUS>DOM | 0.037 | 0.023 | 0.044 |
| 2*Nm* DOM>(CAS, MUS) | 0.043 | 0.002 | 0.071 |
| 2*Nm* (CAS, MUS)>DOM | 0.091 | 0.006 | 0.323 |

The direction of gene flow is indicated by “>”, with gene flow from the subspecies/group listed second into the subspecies listed first.

Supplementary Table 12. $\hat{\theta}_{\pi}$ /$\hat{D}_{xy}$ matrix for the 200 randomly selected autosomal loci from the genomic data.

|  | *M. m. castaneus* | *M. m. domesticus* | *M. m. musculus* |
| --- | --- | --- | --- |
| *M. m. castaneus* | 0.0035 | 0.0044 | 0.0038 |
| *M. m. domesticus* | 0 | 0.0019 | 0.0043 |
| *M. m. musculus* | 0 | 0 | 0.0013 |

Each summary statistic was calculated as mean of the number of base pair differences between pairs of sequences divided by the sum of locus length.

Supplementary Table 13. $\hat{\theta}_{\pi}$ /$\hat{D}_{xy}$ matrix for the 4 autosomal loci from Geraldes *et al.* (2008).

|  | *M. m. castaneus* | *M. m. domesticus* | *M. m. musculus* |
| --- | --- | --- | --- |
| *M. m. castaneus* | 0.0038 | 0.0055 | 0.0057 |
| *M. m. domesticus* | - | 0.0011 | 0.0050 |
| *M. m. musculus* | - | - | 0.0011 |

Each summary statistic was calculated as mean of the number of base pair differences between pairs of sequences divided by the sum of locus lengths.

Supplementary Table 14. $F_{st}$ between subspecies was calculated as the mean across loci. Locus specific values of $F_{st}$ were calculated using pairwise differences between sequences following Hudson, Slatkin, and Maddison (1992).

| Dataset | Subspecies 1 | Subspecies 2 | *F_st_* |
| --- | --- | --- | --- |
| 200 loci | *M. m. castaneus* | *M. m. domesticus* | 0.3914 |
|  | *M. m. castaneus* | *M. m. musculus* | 0.3725 |
|  | *M. m. domesticus* | *M. m. musculus* | 0.6355 |
| 4 autosomal loci Geraldes *et al*. 2008 | *M. m. castaneus* | *M. m. domesticus* | 0.4496 |
|  | *M. m. castaneus* | *M. m. musculus* | 0.4249 |
|  | *M. m. domesticus* | *M. m. musculus* | 0.6674 |

Supplementary Table 15. Phylogeny results from the re-analysis of four autosomal and two X-linked loci included in Geraldes *et al*., (2008).

| Species Tree | Count | Frequency |
| --- | --- | --- |
| (1,(0,2)3)4 | 49,421 | 0.479 |
| (2,(0,1)3)4 | 32,563 | 0.315 |
| (0,(1,2)3)4 | 21,273 | 0.206 |

The three subspecies are numbered from 0 to 2 (corresponding to *M. m. castaneus*, *M. m. domesticus*, and *M. m. musculus,* respectively). Ancestral populations are numbered beginning with 3 and are ordered numerically in time (i.e. 4 is the ancestor of all populations).

Supplementary Table 16. Parameter estimates with 95% confidence limits for the most probable model of three subspecies using four autosomal and two X-linked loci included in Geraldes *et al.* (2008).

| Parameter | MLE | 95%Lo | 95%Hi |
| --- | --- | --- | --- |
| *tµ* CAS, MUS | 0.918 | 0.490 | 2.882 |
| *tµ* DOM, (CAS, MUS) | 1.370 | 0.966 | 3.906 |
| *4Nµ* CAS | 2.135 | 1.505 | 3.155 |
| *4Nµ* DOM | 1.205 | 0.845 | 1.775 |
| *4Nµ* MUS | 0.545 | 0.345 | 0.925 |
| *4Nµ* (CAS, MUS) | 0.825 | 0.115 | 8.975 |
| *4Nµ* (CAS, MUS, DOM) | 8.215 | 2.935 | 9.845 |
| *m/µ* CAS>DOM | 0.072^**^ | 0.017 | 0.191 |
| *m/µ* DOM>CAS | 0.042^*^ | 0.008 | 0.184 |
| *m/µ* CAS>MUS | 0.186^***^ | 0.038 | 0.197 |
| *m/µ* MUS>CAS | 0.200^**^ | 0.036 | 0.197 |
| *m/µ* DOM>MUS | 0.000 | 0.002 | 0.176 |
| *m/µ* MUS>DOM | 0.200^***^ | 0.072 | 0.199 |
| *m/µ* DOM>(CAS, MUS) | 0.200 | 0.006 | 0.196 |
| *m/µ* (CAS, MUS)>DOM | 0.200 | 0.008 | 0.197 |

Subspecies names are abbreviated, CAS for *Mus musculus castaneus*, DOM for *Mus musculus domesticus,* and MUS for *Mus musculus musculus.* For each parameter, the estimated value is the maximum of the estimated marginal posterior probability density for that parameter. Population size and splitting time parameters are shown scaled by *µ*, and migration rate parameters are shown scaled by the inverse of *µ*, where *µ* is the geometric mean of the mutation rates (Hey & Nielsen 2004). The direction of gene flow is indicated by “>”, with gene flow from the subspecies/group listed second into the subspecies listed first (^*^p < 0.05, ^**^p< 0.01, ^***^p < 0.001).

Supplementary Table 17. Estimated split times (years) and effective population sizes (*N_e_*) with 95% confidence limits for an analysis using four autosomal and two X-linked loci included in Geraldes *et al.* (2008).

| Parameter | MLE | 95%Lo | 95%Hi |  |
| --- | --- | --- | --- | --- |
| Time since split between CAS and MUS | 332,950 | 177,719 | 1,045,275 | |
| Time since split between DOM and (CAS, MUS) | 496,887 | 350,359 | 1,416,671 | |
| *N_e_* CAS | 258,115 | 181,950 | 381,430 | |
| *N_e_* DOM | 145,681 | 102,158 | 214,592 | |
| *N_e_* MUS | 65,889 | 41,709 | 111,830 | |
| *N_e_* Ancestral population (CAS, MUS) | 99,740 | 13,903 | 1,085,050 | |
| *N_e_* Ancestral population (CAS, MUS, DOM) | 993,169 | 354,833 | 1,190,231 | |

Estimates assume 1.5 generations/year and $6\times{10}^{-9}$ mutations per base pair per generation.

Supplementary Table 18. Estimated population migration rates with 95% confidence limits given the most probable topology for an analysis using four autosomal and two X-linked loci included in Geraldes *et al.* (2008).

| Value | MLE | 95%Lo | 95%Hi |
| --- | --- | --- | --- |
| 2*Nm* CAS>DOM | 0.0808 | 0.0186 | 0.2214 |
| 2*Nm* CAS>MUS | 0.1485 | 0.0416 | 0.2518 |
| 2*Nm* DOM>CAS | 0.0266 | 0.0052 | 0.1156 |
| 2*Nm* DOM>MUS | 0.0001 | 0.0010 | 0.1064 |
| 2*Nm* DOM>(CAS,MUS) | 0.0810 | 0.0037 | 0.1421 |
| 2*Nm* MUS>CAS | 0.0367 | 0.0104 | 0.0726 |
| 2*Nm* MUS>DOM | 0.0421 | 0.0191 | 0.0776 |
| 2*Nm* (CAS,MUS)>DOM | 0.0005 | 0.0025 | 0.6172 |

The direction of gene flow is indicated by “>”, with gene flow from the subspecies/group listed second into the subspecies listed first.

Supplementary Table 19. Summary information for 200 loci, including location on the chromosome, length, number of variable sites, and recombination rate (cM/Mb). Recombination rates were based on sex-averaged maps (Cox et al. 2009) as reported at <http://cgd.jax.org/mousemapconverter/>.

| Position (Chr:First:Last) | Length | # Polymorphic sites | cM/Mb |
| --- | --- | --- | --- |
| 1:15474901:15474982 | 81 | 3 | 0.6473 |
| 1:20035276:20035461 | 185 | 2 | 0.1486 |
| 1:22573860:22574280 | 420 | 10 | 0.2938 |
| 1:24821991:24822769 | 778 | 24 | 0.4331 |
| 1:51097180:51097438 | 258 | 4 | 0.8885 |
| 1:77047966:77048334 | 368 | 9 | 0.5400 |
| 1:105184131:105184344 | 213 | 5 | 0.3958 |
| 1:108140471:108141567 | 1096 | 22 | 0.1896 |
| 1:142890494:142891721 | 1227 | 40 | 0.0942 |
| 1:144913851:144914143 | 292 | 8 | 0.1971 |
| 1:150932300:150932420 | 120 | 4 | 0.0994 |
| 1:166574679:166575515 | 836 | 29 | 0.9598 |
| 1:175332327:175332379 | 52 | 3 | 0.6981 |
| 1:194245191:194245746 | 555 | 12 | 0.4865 |
| 1:194584858:194585190 | 332 | 10 | 0.2951 |
| 2:8069961:8070974 | 1013 | 21 | 1.4752 |
| 2:26037845:26037895 | 50 | 4 | 0.8478 |
| 2:34323970:34325480 | 1510 | 18 | 0.2324 |
| 2:45247089:45247684 | 595 | 13 | 0.0971 |
| 2:56404841:56405051 | 210 | 5 | 0.4453 |
| 2:68645065:68645969 | 904 | 22 | 0.2392 |
| 2:79176116:79176253 | 137 | 6 | 0.6455 |
| 2:100252235:100253076 | 841 | 27 | 0.2399 |
| 2:107974286:107975086 | 800 | 17 | 0.1923 |
| 2:108693769:108694634 | 865 | 27 | 0.3355 |
| 2:117133499:117134750 | 1251 | 15 | 0.5647 |
| 2:148097851:148097973 | 122 | 3 | 0.0994 |
| 2:152049693:152051003 | 1310 | 23 | 0.5631 |
| 2:161091041:161091340 | 299 | 10 | 1.5271 |
| 2:161133524:161134284 | 760 | 29 | 1.5895 |
| 2:179084543:179084626 | 83 | 3 | 1.4937 |
| 3:3373401:3374875 | 1474 | 24 | 0.3260 |
| 3:5773166:5774208 | 1042 | 18 | 0.0005 |
| 3:37748503:37748884 | 381 | 8 | 0.1472 |
| 3:39106388:39107089 | 701 | 15 | 0.3864 |
| 3:42234775:42235540 | 765 | 13 | 0.4334 |
| 3:61699272:61700119 | 847 | 19 | 0.1439 |
| 3:73258865:73259919 | 1054 | 25 | 1.1874 |
| 3:73514096:73514925 | 829 | 25 | 1.2002 |
| 3:78310718:78311813 | 1095 | 25 | 0.0948 |
| 3:110064699:110065296 | 597 | 17 | 0.1456 |
| 3:111375829:111376670 | 841 | 24 | 0.3838 |
| 3:142226166:142226801 | 635 | 12 | 0.5815 |
| 3:149386433:149386843 | 410 | 12 | 3.0866 |
| 4:4668485:4669665 | 1180 | 28 | 0.5193 |
| 4:11475392:11475577 | 185 | 4 | 0.6440 |
| 4:22300768:22301426 | 658 | 7 | 0.9681 |
| 4:26735377:26736588 | 1211 | 31 | 0.4243 |
| 4:28032645:28033101 | 456 | 16 | 0.9288 |
| 4:29161979:29162126 | 147 | 9 | 0.8934 |
| 4:35471824:35472115 | 291 | 8 | 1.1335 |
| 4:87460354:87460777 | 423 | 16 | 0.4407 |
| 4:87905552:87905583 | 31 | 2 | 0.5491 |
| 4:96979112:96979453 | 341 | 6 | 0.5899 |
| 4:114521936:114522231 | 295 | 8 | 0.2956 |
| 5:5975767:5975992 | 225 | 6 | 0.3461 |
| 5:7592152:7592400 | 248 | 10 | 0.0494 |
| 5:9664223:9664339 | 116 | 3 | 0.0994 |
| 5:11945547:11945935 | 388 | 5 | 0.0981 |
| 5:26211635:26211729 | 94 | 3 | 7.2157 |
| 5:42623005:42623609 | 604 | 29 | 0.3883 |
| 5:46316115:46316442 | 327 | 4 | 0.1476 |
| 5:50378300:50378548 | 248 | 6 | 0.0494 |
| 5:51811228:51812981 | 1753 | 36 | 0.8734 |
| 5:58329810:58330639 | 829 | 17 | 0.5281 |
| 5:61138546:61138917 | 371 | 12 | 0.1473 |
| 5:67209528:67209673 | 145 | 6 | 0.2978 |
| 5:90119328:90119660 | 332 | 7 | 0.3443 |
| 6:3949117:3949380 | 263 | 8 | 0.4441 |
| 6:9982019:9983039 | 1020 | 16 | 0.2854 |
| 6:16137381:16137457 | 76 | 2 | 0.6973 |
| 6:46257184:46257680 | 496 | 13 | 0.1952 |
| 6:51896116:51896323 | 207 | 5 | 0.9402 |
| 6:51975154:51976606 | 1452 | 20 | 0.9323 |
| 6:53292148:53293047 | 899 | 13 | 0.1914 |
| 6:58526640:58527421 | 781 | 16 | 0.5774 |
| 6:61493016:61493288 | 272 | 9 | 0.1480 |
| 6:77350851:77352072 | 1221 | 32 | 1.6492 |
| 6:101970020:101970503 | 483 | 12 | 0.2441 |
| 6:102298909:102299225 | 316 | 8 | 0.1969 |
| 6:127834071:127834230 | 159 | 2 | 1.4385 |
| 6:147424516:147424783 | 267 | 8 | 0.5427 |
| 7:53576829:53577040 | 211 | 6 | 0.0990 |
| 7:62808916:62809660 | 744 | 13 | 0.1446 |
| 7:70598902:70599240 | 338 | 4 | 3.8350 |
| 7:102822349:102823128 | 779 | 28 | 0.1444 |
| 7:129360103:129360461 | 358 | 14 | 1.7191 |
| 8:6125598:6125975 | 377 | 10 | 0.1963 |
| 8:7759741:7759982 | 241 | 7 | 0.3458 |
| 8:14558124:14558888 | 764 | 9 | 0.7705 |
| 8:17518644:17519457 | 813 | 17 | 0.8168 |
| 8:30993416:30993665 | 249 | 6 | 1.6790 |
| 8:32500819:32501417 | 598 | 19 | 0.4369 |
| 8:49230344:49230606 | 262 | 4 | 1.3325 |
| 8:49729660:49730080 | 420 | 6 | 0.8325 |
| 8:53468191:53469004 | 813 | 18 | 0.0961 |
| 8:57686097:57686247 | 150 | 7 | 1.3399 |
| 8:73599456:73600228 | 772 | 15 | 0.2407 |
| 8:76309648:76309736 | 88 | 3 | 0.6969 |
| 8:78155627:78155726 | 99 | 3 | 0.5970 |
| 8:80240052:80240281 | 229 | 6 | 0.5932 |
| 8:87817901:87818347 | 446 | 9 | 0.5869 |
| 8:120409124:120409807 | 683 | 13 | 1.8855 |
| 9:13888833:13888997 | 164 | 12 | 0.8430 |
| 9:28153193:28153877 | 684 | 16 | 0.0967 |
| 9:33418988:33419795 | 807 | 28 | 0.2884 |
| 9:33685444:33685732 | 288 | 14 | 0.2957 |
| 9:49612414:49613315 | 901 | 26 | 0.2871 |
| 9:71069003:71069254 | 251 | 6 | 0.3456 |
| 9:86950408:86950501 | 93 | 7 | 0.2986 |
| 9:90490924:90491189 | 265 | 2 | 0.6908 |
| 10:9100835:9101714 | 879 | 22 | 0.7184 |
| 10:15550403:15550620 | 217 | 7 | 0.6925 |
| 10:22961609:22961796 | 187 | 11 | 0.1981 |
| 10:28455711:28455781 | 70 | 3 | 1.7438 |
| 10:113531569:113532172 | 603 | 21 | 0.5339 |
| 11:19159776:19160144 | 368 | 14 | 1.1783 |
| 11:36584179:36584790 | 611 | 9 | 0.9703 |
| 11:36791215:36791283 | 68 | 2 | 0.9467 |
| 11:37511006:37512050 | 1044 | 15 | 1.9482 |
| 11:39772973:39774124 | 1151 | 19 | 0.5673 |
| 11:41636560:41637262 | 702 | 16 | 0.6762 |
| 11:44724890:44724958 | 68 | 3 | 2.3917 |
| 11:44896961:44897744 | 783 | 19 | 0.2887 |
| 11:47241473:47242455 | 982 | 34 | 0.2383 |
| 11:68143988:68144384 | 396 | 4 | 0.8335 |
| 11:76712466:76712555 | 89 | 4 | 0.2987 |
| 11:82576261:82576417 | 156 | 4 | 0.2977 |
| 12:7541650:7542167 | 517 | 14 | 0.1462 |
| 12:9276395:9276645 | 250 | 9 | 0.0494 |
| 12:9390099:9390762 | 663 | 17 | 0.0484 |
| 12:10442430:10443565 | 1135 | 25 | 1.0882 |
| 12:10666243:10666522 | 279 | 5 | 0.1479 |
| 12:29215507:29215612 | 105 | 4 | 0.5471 |
| 12:34203743:34204913 | 1170 | 17 | 0.7085 |
| 12:46598574:46599054 | 480 | 13 | 0.1465 |
| 12:52451755:52452308 | 553 | 15 | 0.0487 |
| 12:56517352:56517695 | 343 | 3 | 0.4424 |
| 12:58342866:58342918 | 52 | 4 | 0.7979 |
| 12:69537455:69537952 | 497 | 11 | 0.1464 |
| 12:70755303:70756506 | 1203 | 27 | 0.2830 |
| 12:89118773:89118872 | 99 | 3 | 1.3930 |
| 12:89950501:89951009 | 508 | 12 | 0.3413 |
| 12:90613059:90613902 | 843 | 21 | 0.3358 |
| 12:95652156:95652715 | 559 | 15 | 0.4864 |
| 13:16279205:16279746 | 541 | 12 | 0.3408 |
| 13:17141765:17142580 | 815 | 19 | 0.1441 |
| 13:29611694:29611789 | 95 | 4 | 0.1990 |
| 13:37312221:37313108 | 887 | 15 | 2.0107 |
| 13:49845544:49846518 | 974 | 18 | 0.3814 |
| 13:70096507:70097058 | 551 | 8 | 0.2919 |
| 13:106329426:106330169 | 743 | 18 | 1.2052 |
| 13:109363729:109363784 | 55 | 3 | 0.2493 |
| 14:15777816:15777917 | 101 | 2 | 0.0497 |
| 14:25179637:25180161 | 524 | 11 | 0.8283 |
| 14:28318177:28318576 | 399 | 7 | 0.1961 |
| 14:28488937:28489221 | 284 | 9 | 0.1972 |
| 14:64059343:64059809 | 466 | 11 | 0.0977 |
| 14:80778914:80779176 | 262 | 8 | 0.1481 |
| 14:86577839:86578484 | 645 | 16 | 0.3390 |
| 14:86700699:86701662 | 963 | 12 | 0.2862 |
| 14:98368592:98369395 | 803 | 23 | 0.7210 |
| 14:100898754:100899276 | 522 | 16 | 0.8283 |
| 14:104330934:104331931 | 997 | 23 | 1.2382 |
| 14:113074774:113076148 | 1374 | 32 | 0.5614 |
| 15:5836024:5836792 | 768 | 31 | 0.0481 |
| 15:22151709:22152656 | 947 | 16 | 0.6683 |
| 15:24778236:24779090 | 854 | 17 | 0.2398 |
| 15:40976624:40976738 | 114 | 2 | 0.5966 |
| 15:45830877:45831749 | 872 | 20 | 0.3833 |
| 15:56717368:56718054 | 686 | 11 | 0.9668 |
| 15:63601811:63602058 | 247 | 11 | 2.3212 |
| 15:68759886:68760850 | 964 | 23 | 1.4787 |
| 16:8001347:8002378 | 1031 | 14 | 0.4279 |
| 16:28484426:28484562 | 136 | 3 | 0.3973 |
| 16:39193407:39193440 | 33 | 2 | 0.3993 |
| 16:39862605:39863446 | 841 | 25 | 0.2879 |
| 16:40404133:40404553 | 420 | 6 | 0.3918 |
| 16:64054193:64054718 | 525 | 11 | 0.4872 |
| 16:68604534:68605344 | 810 | 14 | 0.1442 |
| 17:11976830:11977227 | 397 | 11 | 0.4902 |
| 17:45084191:45085077 | 886 | 19 | 1.1969 |
| 17:60576236:60577068 | 832 | 32 | 0.4800 |
| 17:62906863:62907010 | 147 | 4 | 0.1489 |
| 17:77649535:77649730 | 195 | 8 | 0.4951 |
| 17:89732832:89733607 | 775 | 18 | 1.1552 |
| 17:93945669:93947083 | 1414 | 23 | 0.6537 |
| 18:4579178:4580208 | 1030 | 26 | 0.5706 |
| 18:25742163:25742334 | 171 | 6 | 1.5864 |
| 18:27866961:27867465 | 504 | 6 | 0.8291 |
| 18:37125975:37126268 | 293 | 7 | 0.0986 |
| 18:54787481:54787601 | 120 | 7 | 0.1491 |
| 18:80824635:80825375 | 740 | 16 | 0.4821 |
| 18:83713083:83713293 | 210 | 5 | 0.9401 |
| 18:89904007:89904237 | 230 | 6 | 0.6426 |
| 19:13717768:13718882 | 1114 | 31 | 0.2368 |
| 19:15543241:15543641 | 400 | 14 | 1.1764 |
| 19:26244839:26245047 | 208 | 8 | 0.2474 |
| 19:28337643:28337898 | 255 | 3 | 1.0367 |
| 19:28560418:28560488 | 70 | 3 | 1.0463 |


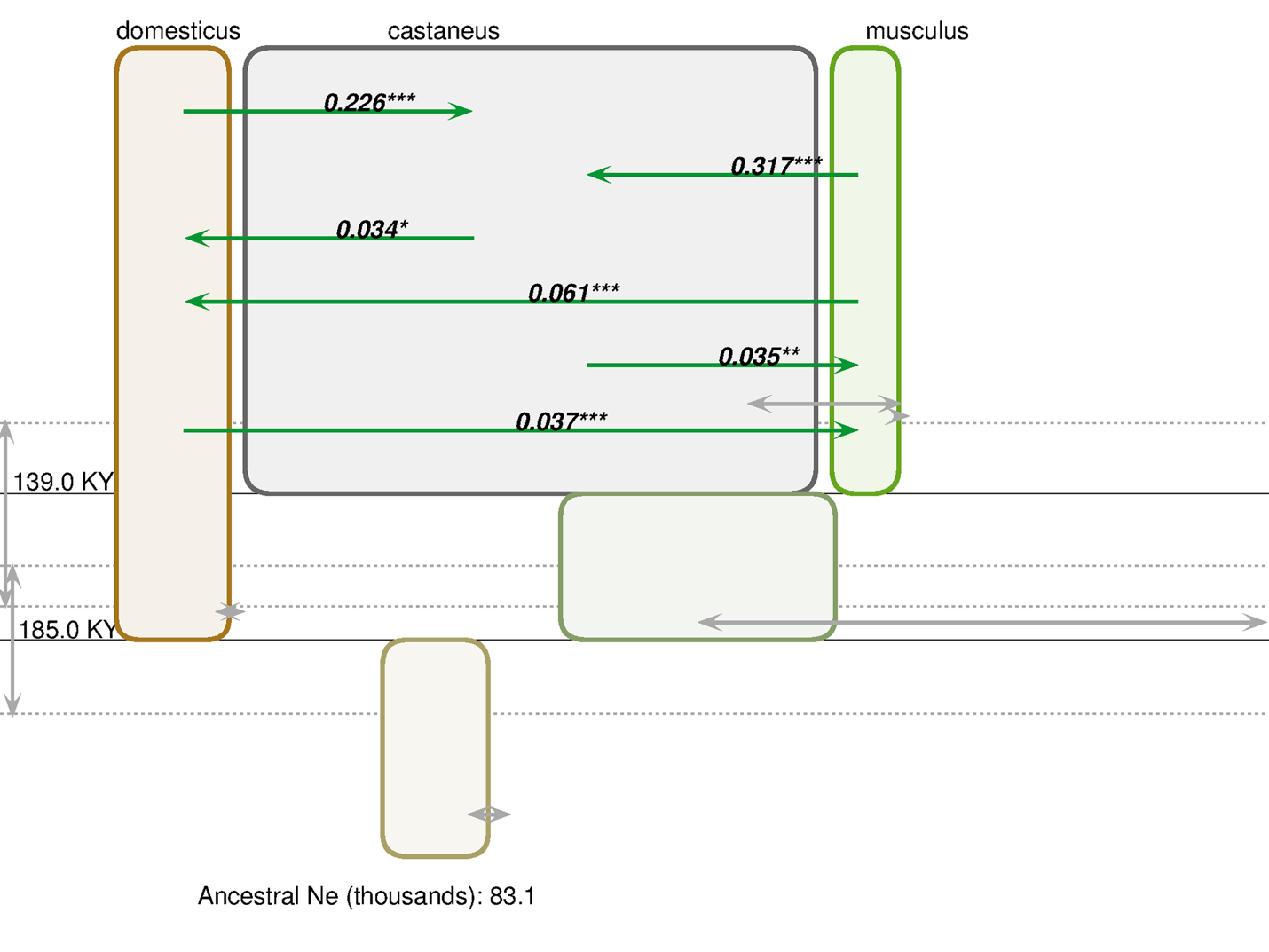


Supplementary Figure 1. A representation of an estimated Isolation with Migration model generated by IMa3 and the IMfig program (Hey *et al*. 2018) for house mice using a set of 200 alternative random autosomal loci. Details are as given in Figure 1. Estimates assume 0.75 generations/year and $6\times{10}^{-9}$ mutations per base pair per generation.


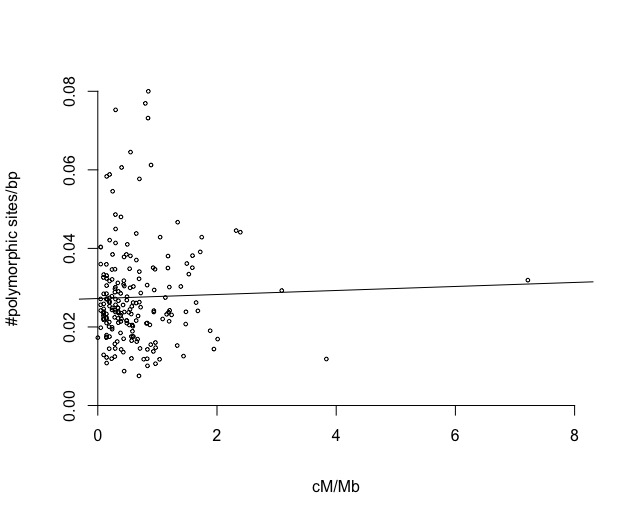


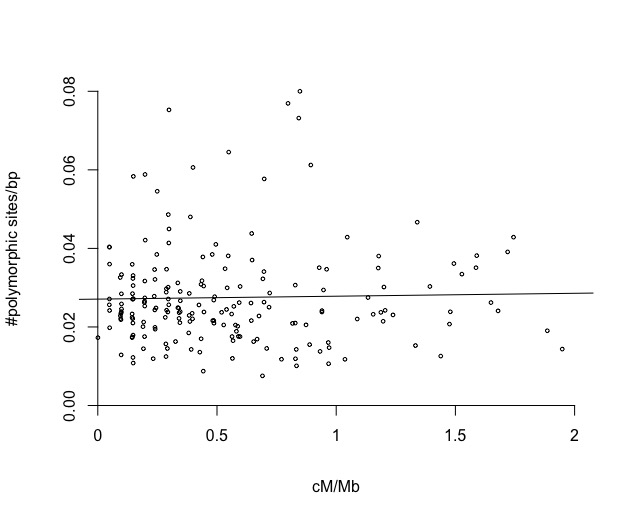


Supplementary Figure 2. SNP density plotted against recombination rate for 200 sampled loci for **A)** all loci ($y=0.0005x + 0.0272$, R^2^ = 0.0009) and **B)** all loci excluding outliers with recombination rate greater than 2 cM/Mb ($y=0.0008x+0.271$, R^2^ = 0.0007; data from Table S19).
